# Chronic Musculoskeletal Pain as an Initial Presentation of Gitelman Syndrome in Adulthood: A Case Report

**DOI:** 10.7759/cureus.110572

**Published:** 2026-06-10

**Authors:** Shreya Nair, Lisha Michel M, Kiran Kumar, Nainika M Fathima, Hygieia R Jacob

**Affiliations:** 1 Internal Medicine, Gulf Medical University, Ajman, ARE; 2 Internal Medicine, Thumbay Group, Ajman, ARE; 3 Internal Medicine, Gulf Medical University, Dubai, ARE

**Keywords:** gitelman syndrome, hypocalciuria, hypokalemia, hypomagnesaemia, metabolic alkalosis, musculoskeletal pains

## Abstract

Gitelman syndrome is an autosomal recessive salt-wasting tubulopathy characterized by hypokalemia, metabolic alkalosis, hypomagnesaemia and hypocalciuria. It may present in adulthood with nonspecific symptoms including cramps, fatigue and musculoskeletal complaints.

We report the case of a 34-year-old male electrical engineer with a three-year history of low back pain, neck and shoulder pain, and radiculopathy. His spinal history is briefly noted as contextual. He was found to have persistent hypokalemia (serum K⁺ ~2.9-3.4 mmol/L), hypomagnesaemia (serum Mg²⁺ ~0.97-1.16 mmol/L), metabolic alkalosis (serum HCO₃⁻ ~28-31 mmol/L, arterial blood gas pH ~7.49) and low urinary fractional excretion of calcium (FECa ~0.002). Work-up excluded other causes of potassium and magnesium wasting; normal blood pressure was noted. A clinical diagnosis of Gitelman syndrome was made. Management included counselling regarding a high-sodium diet together with potassium-rich and magnesium-rich foods, supplementation of potassium and magnesium, and planned initiation of eplerenone and sodium chloride supplementation.

We discuss the pathophysiology of Gitelman syndrome, its typical biochemical profile, differential diagnosis (including Bartter syndrome), the relevance of the patient’s musculoskeletal pain in the setting of electrolyte imbalance, and therapeutic considerations.

This case underscores the importance of considering Gitelman syndrome in adults presenting with persistent hypokalemia, hypomagnesaemia and metabolic alkalosis, even when musculoskeletal symptoms dominate the presentation. Early recognition allows targeted therapy, potentially improving quality of life.

## Introduction

Gitelman syndrome (GS) is a hereditary renal tubulopathy characterized by renal salt-wasting due to loss-of-function of the thiazide-sensitive sodium-chloride co-transporter (NCC) in the distal convoluted tubule (DCT) [1,2]. Typical biochemical features include hypokalemia, metabolic alkalosis, hypomagnesaemia and low urinary calcium excretion (hypocalciuria) [3]. The estimated prevalence is approximately one in 40,000 individuals, although the phenotype is variable and diagnosis may be delayed into adulthood [3]. Patients may present with fatigue, muscle cramps, tetany, and in some cases, musculoskeletal complaints owing to electrolyte disturbances [3]. The co-existence of persistent musculoskeletal pain may obscure the underlying renal tubular disorder, as in our patient whose primary complaints were low back, cervical and shoulder pain. We report a case of GS in a 34-year-old male, highlighting the diagnostic process and management considerations.

## Case presentation

A 34-year-old male electrical engineer presented in September 2025 with persistent neck and right shoulder pain, stiffness, and chronic low back discomfort. His symptoms dated back more than three years to a work-related lifting injury, after which he developed low back pain with radiculopathy. Approximately two years later, he began experiencing neck pain and right-sided shoulder pain with associated stiffness. Despite multiple interventions, including analgesics, physiotherapy, chiropractic care, epidural steroid injections, ozone therapy, facet-joint injections, and laser spinal treatment, his symptoms persisted. He reported no current numbness or tingling. There was no known family history of renal tubular disorders. On examination, neck and spinal movements were non-tender and without restriction. The patient was normotensive, denied diuretic use, and had an unremarkable neurological examination.

Key laboratory findings are summarized in Table 1 with relevant dates.

**Table 1:** Laboratory findings ABG: Arterial Blood Gas

Date	Parameter	Value	Unit	Reference Range
Day 1	Random Blood Sugar	113	mg/dL	70-110 mg/dL
	Uric Acid	8.5	mg/dL	3.5-7.0 mg/dL
	Total Calcium	9.37	mg/dL	8.60-10.0 mg/dL
	Serum Creatinine	0.88	mg/dL	0.7-1.3 mg/dL
	Serum Magnesium (Mg^2+^)	0.97	mmol/L	0.70-1.10 mmol/L
	Serum Potassium (K^+^)	2.9	mmol/L	3.5-5.1 mmol/L
	Serum Chloride (Cl^-^)	96	mmol/L	98-107 mmol/L
	Serum Bicarbonate (HCO₃⁻)	28.5	mmol/L	22-28 mmol/L
Day 4	ABG: pH	7.49		7.35-7.45
	ABG: HCO₃⁻	31.2	mmol/L	22-28 mmol/L
Day 15	Ca²⁺ (Urine) - 24 Hour	1.7	mmol/24h	<2.5 mmol/24h
Day 30	FECa^2+^ (Fractional Excretion of Calcium)	0.002		<0.02 (low)
Day 180	Aldosterone	9	ng/dL	
	Renin	0.24	ng/mL/hr	
	Aldosterone/Renin Ratio	37.5		<20

In view of persistent hypokalemia, hypomagnesaemia, metabolic alkalosis, in the context of normal blood pressure and low urinary calcium excretion and elevated aldosterone to renin ratio, a clinical diagnosis of GS was made. Patient was counselled on a high-sodium, high-potassium and high-magnesium diet with avoidance of strenuous exercise given muscular symptoms. Genetic testing (SLC12A3 mutation) was advised however could not be performed due to lack of availability.

Patient was also started on oral magnesium and potassium replacement. He was advised to come for close follow-up for repeat testing. Follow-up laboratory evaluation after a month demonstrated slight improvement in potassium and magnesium levels. Serum potassium rose up to 3.4 mmol/L and magnesium to 1.12 mmol/L. Patient however continued to have persistent symptoms.

Despite ongoing supplementation, correction of electrolyte levels remained suboptimal, largely due to poor tolerance to higher doses of oral potassium. Also on repeat testing, the magnesium levels again came down to 1.0 mmol/L. In view of persistent renal potassium wasting, the patient was counselled regarding the role of potassium-sparing agents in the management of GS, and therapy with eplerenone was initiated while continuing oral potassium and magnesium supplementation, with plans for close biochemical and clinical follow-up.

As of now, patient continues to be on high-dose magnesium (magnesium bisglycinate) replacement (800 mgs thrice a day), potassium replacement (600 mgs by mouth once daily) and tab eplerenone 25 mgs by mouth once daily. The most recent test done during the last follow-up two weeks ago showed magnesium levels of 1.0 mmol/L and potassium of 3.0 mmol/L. In view of persistent hypomagnesemia, patient has been advised to take injection magnesium sulphate 2 grams as IV infusion over one hour on an outpatient basis and has been advised close follow-up. 

## Discussion

This case illustrates typical features of GS in adulthood: persistent hypokalemia, hypomagnesaemia, metabolic alkalosis, low urinary fractional excretion of calcium (FECa ~0.002), normo-tension and nonspecific symptoms [1,2,4]. GS is caused by bi-allelic inactivating mutations in the SLC12A3 gene encoding NCC in the DCT, resulting in impaired NaCl reabsorption, increased distal Na⁺ delivery, enhanced K⁺ and H⁺ secretion and activation of the renin-angiotensin-aldosterone system (RAAS) [1-3]. The renal phenotype thus includes renal potassium wasting, hypokalemia, metabolic alkalosis, hypomagnesaemia (impaired Mg²⁺ reabsorption in DCT) and hypocalciuria (increased calcium reabsorption secondary to reduced NaCl uptake) [3,5].

Our patient’s musculoskeletal symptoms (low back, shoulder, neck pain, stiffness and radiculopathy) are not classic for GS, but the literature does support that electrolyte abnormalities in GS may lead to fatigue, muscle cramps, tetany, and musculoskeletal discomfort [4]. While his predominant complaint was spinal and shoulder pain (probably from disc disease and injury), his electrolyte disturbances may have contributed to the persistence of muscle-related symptoms and stiffness, highlighting the importance of considering an underlying systemic cause [5]. Differential diagnosis includes adult-onset Bartter syndrome (Type 3), which can present later and may not always have hypercalciuria. GS generally presents later, is milder, and is distinguished by hypomagnesaemia and hypocalciuria [6,7].

Management of GS is largely supportive and symptomatic: liberal salt intake, potassium and magnesium supplementation, and in some cases use of potassium-sparing agents such as spironolactone or eplerenone, or non-steroidal anti-inflammatory drugs (in Bartter, less so in GS) [7,8]. Lifelong supplementation may be required; prognosis is generally good, but quality of life may be impaired [7,8].

In this case, the very low urinary calcium excretion (FECa ~0.002) (hypocalciuria) supports the diagnosis of GS [3]. The absence of family history or genetic confirmation is a limitation, but clinical and biochemical criteria strongly point to GS. Continued follow-up, genetic testing and optimization of electrolyte supplementation are key [4,6,7].

## Conclusions

In adults presenting with persistent hypokalemia, hypomagnesaemia, metabolic alkalosis and low urinary calcium excretion, GS should be strongly considered, even in the presence of musculoskeletal pain as a prominent symptom. Recognition enables targeted therapy (dietary and pharmacologic), which may improve symptoms and prevent complications. This case underscores the need for a high index of suspicion in “non-classical” presentations.
